# A g-Type Lysozyme from Deep-Sea Hydrothermal Vent Shrimp Kills Selectively Gram-Negative Bacteria

**DOI:** 10.3390/molecules26247624

**Published:** 2021-12-16

**Authors:** Jing-Chang Luo, Jian Zhang, Li Sun

**Affiliations:** 1 CAS and Shandong Province Key Laboratory of Experimental Marine Biology, Center for Ocean Mega-Science, Institute of Oceanology, Chinese Academy of Sciences, Qingdao 266071, China; luojingchang@qdio.ac.cn; 2 Laboratory for Marine Biology and Biotechnology, Pilot National Laboratory for Marine Science and Technology (Qingdao), Qingdao 266200, China; 3 College of Earth and Planetary Sciences, University of Chinese Academy of Sciences, Beijing 100049, China; 4 School of Ocean, Yantai University, Yantai 264005, China; zhangjian@ytu.edu.cn

**Keywords:** g-type lysozyme, shrimp, deep-sea hydrothermal vent, non-enzymatic antibacterial activity

## Abstract

Lysozyme is a key effector molecule of the innate immune system in both vertebrate and invertebrate. It is classified into six types, one of which is the goose-type (g-type). To date, no study on g-type lysozyme in crustacean has been documented. Here, we report the identification and characterization of a g-type lysozyme (named LysG1) from the shrimp inhabiting a deep-sea hydrothermal vent in Manus Basin. LysG1 possesses conserved structural features of g-type lysozymes. The recombinant LysG1 (rLysG1) exhibited no muramidase activity and killed selectively Gram-negative bacteria in a manner that depended on temperature, pH, and metal ions. rLysG1 bound target bacteria via interaction with bacterial cell wall components, notably lipopolysaccharide (LPS), and induced cellular membrane permeabilization, which eventually caused cell lysis. The endotoxin-binding capacity enabled rLysG1 to alleviate the inflammatory response induced by LPS. Mutation analysis showed that the bacterial binding and killing activities of rLysG1 required the integrity of the conserved α3 and 4 helixes of the protein. Together, these results provide the first insight into the activity and working mechanism of g-type lysozyme in crustacean and deep-sea organisms.

## 1. Introduction

The innate immune system is an important mechanism of the host to defend against pathogens, especially for invertebrate, which lack adaptive immune systems. Lysozyme (muramidase, EC 3.2.1.17) is one of the key molecules of the innate immune system. It catalyzes the cleavage of the beta-1,4-glycosidic bond between the C-1 of N-acetylmuramic acid (MurNAc) and the C-4 of N-acetylglucosamine (GlcNAc) in peptidoglycan [1], an essential component of the bacterial cell wall. As a result, lysozyme is widely employed by animals as a weapon against bacterial infection. Since, compared to Gram-negative bacteria, Gram-positive bacteria have a peptidoglycan cell wall that is much thicker and exposed to the extracellular milieu, lysozyme is generally more efficient at killing Gram-positive bacteria. However, some lysozymes are known to kill effectively Gram-negative bacteria [2,3,4,5]. In recent years, lysozyme has been reported to have functions other than muramidase, such as isopeptidase, chitinase, and non-enzyme antibacterial activities [5]. In some cases, the mechanism of the non-enzymatic antibacterial effect was attributed to the extreme cationic nature of the lysozymes [6,7]. In addition to its antibacterial activity, lysozyme has an influence on the inflammatory response [8,9]. For example, hen egg lysozyme (HEL) can alleviate the immune response to inflammatory bowel disease [10], and human lysozyme can affect inflammation by regulating serum complement activation [11].

According to their difference in source, amino acid sequence, chemical property, and biological activity, lysozymes are classified into six types, including chicken-type (c-type), goose-type (g-type), plant-type, and invertebrate-type (i-type) [12]. The g-type lysozyme was initially identified from egg white in 1967 [13]. Since then, g-type lysozymes from a variety of vertebrate species have been studied in the aspects of molecular evolution, structure, and function [14,15]. In contrast, g-type lysozyme has been reported only in a small number of chordate and mollusk [16,17,18,19,20]. In crustaceans, several c-type and i-type lysozymes have been identified and characterized, some of which showed microbicidal activity [2,5,21,22,23,24,25,26,27]. However, to our knowledge, no study on crustacean g-type lysozyme has been documented.

In this study, we identified a g-type lysozyme (named LysG1) from the *Alvinocarididae* shrimp (*Rimicaris* sp.) collected from a deep sea hydrothermal vent in Desmos, Manus Basin, at ~1883 m depth [28]. In deep sea hydrothermal vents, the ecosystem is constituted mainly by chemoautotrophic bacteria, which are the primary producer that manufacture organic matter through chemosynthesis, and invertebrate animals, notably crustaceans (shrimp and crab) [29,30,31]. The *Alvinocarididae* family of shrimp is known to be a dominant organism in the communities of various hydrothermal vents [32]. To date, several innate immune system factors, i.e., lectin and crustin, have been identified in hydrothermal shrimp [33,34,35], but no lysozyme has been reported. In this study, we investigated the biological characteristics and antimicrobial activity of the g-type lysozyme LysG1. Our results provide insights into the characteristics and biological activity of lysozyme in deep sea hydrothermal vent crustacean. 

## 2. Results 

### 2.1. LysG1 Possesses the Conserved Structure Features of g-Type Lysozyme

**Figure 1:**
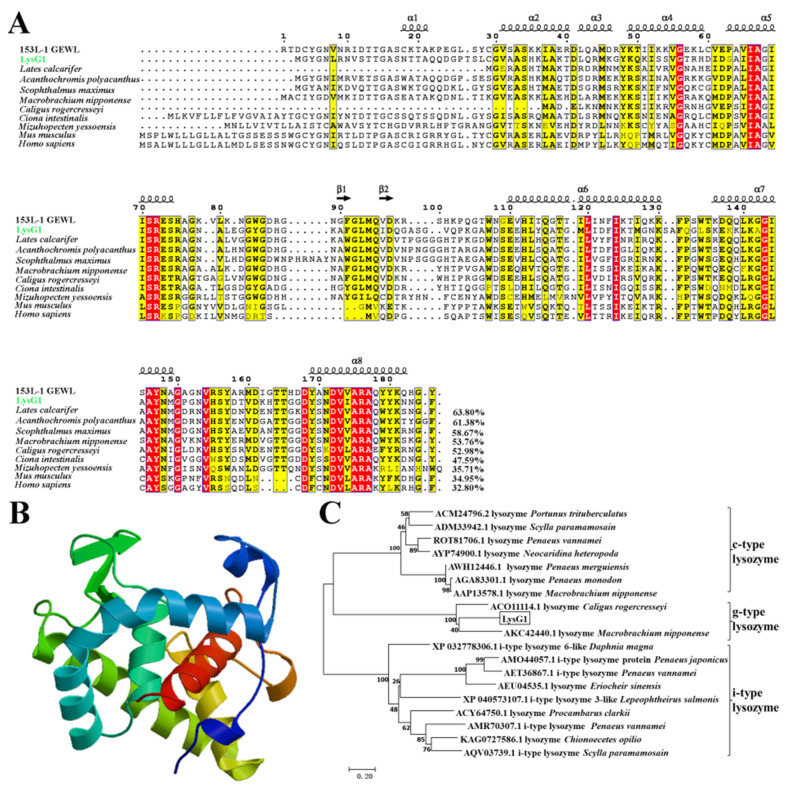
Sequence, phylogenetic, and structural analysis of LysG1. (**A**) Alignment of LysG1 with g-type lysozymes from different species. Dots denote gaps introduced for maximum matching. The consensus residues are in red shade, the residues that are ≥75% identical among the aligned sequences are boxed and highlighted in yellow. The secondary structures (α-helices and β-strands) based on 153L-1 GEWL (Anser) are shown on the top of the aligned sequences. The GenBank accession numbers of the aligned sequences are as follows: 153L-1 GEWL, AEC34022.1; *Lates calcarifer*, XP_018520541.1; *Caligus rogercresseyi*, ACO11114.1; *Macrobrachium nipponense*, AKC42440.1; *Mizuhopecten yessoensis*, AEY77130.1; *Ciona intestinalis*, AOT06085.1; *Scophthalmus maximus*, ADN06441.1; *Mus musculus*, NP_081387.1; *Homo sapiens*, NP_777558.1. (**B**) The predicted structure of LysG1 was built with SWISS-MODEL. (**C**) Phylogenetic analysis of LysG1 homologues in crustaceans. The phylogenetic tree was constructed with MEGA 7.0 using the neighbor-joining method. Numbers beside the internal branches indicate bootstrap values based on 1000 replications.

LysG1 was identified in the shrimp *Rimicaris sp*. from a hydrothermal vent in Desmos, Manus basin. LysG1 protein is composed of 188 amino acid residues, with a calculated molecular weight of 20.0 kDa and a predicted pI of 9.06. It contains the conserved residues of the sugar binding site (residues E72, 94QID96, F124, 150YN151, and G153) and catalytic site (E72 and D96) of g-type lysozyme. LysG1 was predicted to form 8 α-helixes and 2 β-pleated sheets (Figure 1A,B). LysG1 is homologous to the g-type lysozymes of crustacean, teleost, and mammal, and shares the highest sequence identity (63.8%) with the g-type lysozyme of *Lates calcarifer* (Figure 1A). To date, no study on crustacean g-type lysozyme has been reported, however, two g-type lysozyme sequences from *Caligus rogercresseyi* and *Macrobrachium nipponense* (Accession no. ACO11114.1 and AKC42440.1, respectively) have been deposited in GenBank, which are 52.98% and 53.76%, respectively, identical to LysG1 (Figure 1A). Phylogenetic analysis of the crustacean lysozymes showed that LysG1 was grouped into the clade of g-type lysozyme and clustered together with the g-type lysozyme of *M. nipponense* (Figure 1C). 

### 2.2. Recombinant LysG1 (rLysG1) Kills Selectively Gram-Negative Bacteria in a Regulated Manner

**Figure 2:**
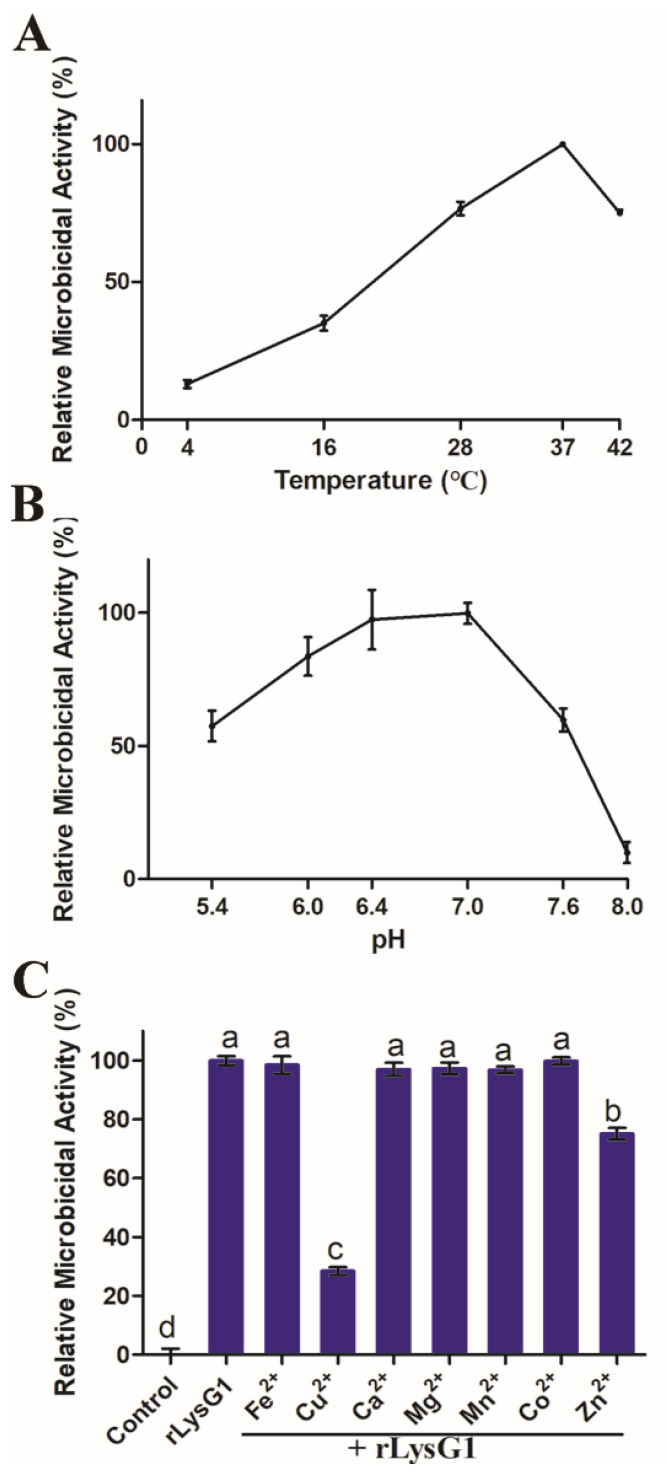
The effect of temperature, pH, and metal ions on the microbicidal activity of rLysG1. (**A**,**B**) rLysG1 was incubated with Escherichia coli at various temperature (**A**) or pH (**B**) for 4 h, and the bactericidal activity of rLysG1 was determined. (**C**) *E. coli* was incubated with rLysG1 or PBS in the presence of different metal ions for 4 h, and the bactericidal activity of rLysG1 was determined. Values are shown as means ± SD (*n* = 3). *n*, the number of replicates. Different letters (a, b, c, and d) indicate statistical significance.

To facilitate study, rLysG1 was prepared (Figure S1). rLysG1 was not able to digest peptidoglycan (PGN) (Figure S2). However, rLysG1 exhibited apparent killing effect on a variety of Gram-negative bacteria. The minimal bactericidal concentration (MBC) of rLysG1 against *Pseudomonas fluorescens*, *Escherichia coli*, *Edwardsiella tarda*, and *Psedoalteromonas hodoensis* were 8, 4, 16, and 4 μM, respectively (Table 1). rLysG1 failed to kill other Gram-negative bacteria, e.g., *Vibrio anguillarum* and *Vibrio harveyi* (Table 1). Likewise, rLysG1 showed no apparent effect on the survival of Gram-positive bacteria from various sources (Table 1). The bactericidal activity of rLysG1 on Gram-negative bacteria was dependent on temperature and pH, and was most efficient at 37 °C and pH 6.4–7.0 (Figure 2A,B). The metal ions Fe^2+^, Ca^2+^, Mg^2+^, Mn^2+^, and Co^2+^ had no significant effect on rLysG1 activity, however, Cu^2+^ and Zn^2+^ inhibited the bactericidal activity of rLysG1 to significant extents (Figure 2C). 

### 2.3. rLysG1 Binds Target Bacteria via Interaction with LPS and Attenuates LPS-Induced Inflammatory Response

**Figure 3:**
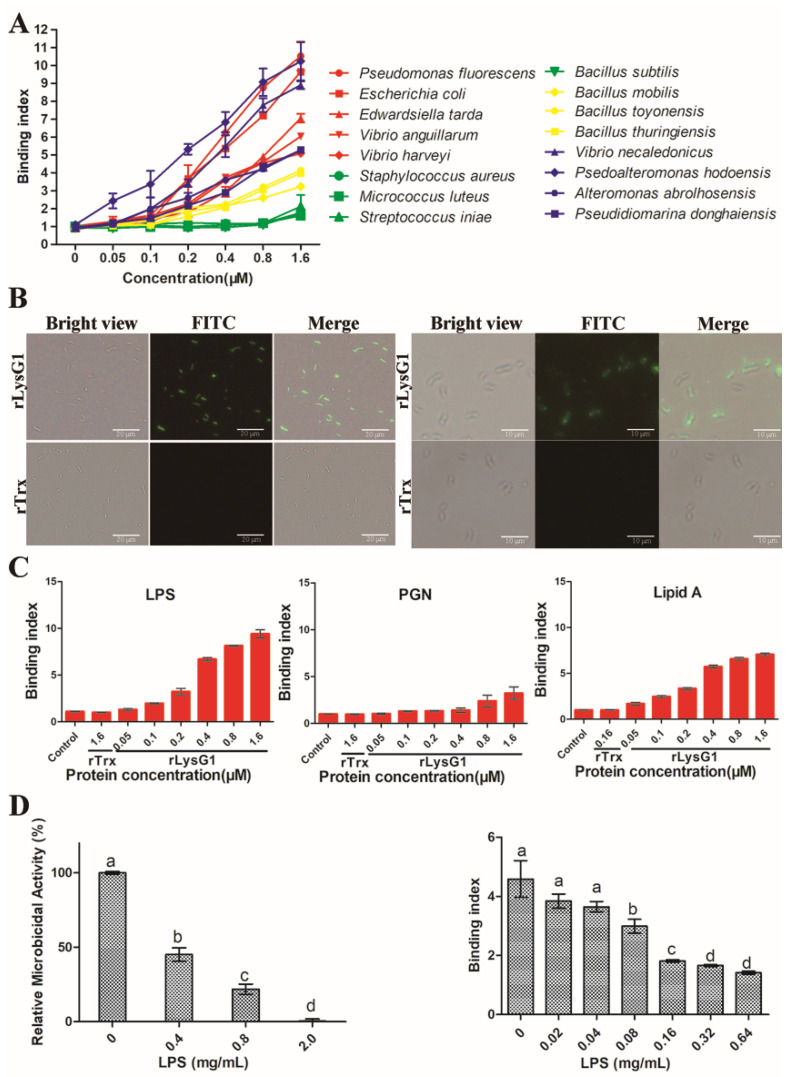
Binding of rLysG1 to bacteria and bacterial cell wall components. (**A**) Bacteria were incubated with different concentrations of rLysG1, and the bound rLysG1 was detected by ELISA. (**B**) *Escherichia coli* (**left**) and *Psedoalteromonas hodoensiswas* (**right**) were incubated with rLysG1 or rTrx. The cells were treated with anti-His antibody and FITC-labeled secondary antibody, and then observed with a fluorescence microscope. (**C**) LPS, PGN and lipid A were incubated with different concentrations of rLysG1, rTrx, or PBS (control), and the protein-bound molecules were detected by ELISA. (**D**) rLysG1 was pre-incubated with different concentrations of LPS and then with *E. coli*. The binding activity (**left**) and bactericidal activity (**right**) of rLysG1 were then determined. For panels (**A**, **C**, and **D**), values are shown as means ± SD (*n* = 3). *n*, the number of replicates. Different letters (a, b, c, and d) indicate statistical significance.

**Figure 4:**
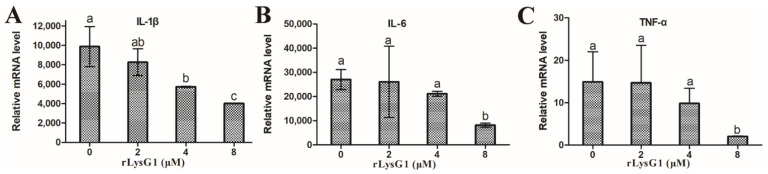
The effect of rLysG1 on expression of inflammatory cytokines in LPS-stimulated RAW264.7 cells. RAW264.7 cells were treated with LPS in the absence or presence of different concentrations of rLysG1, and the expression of IL-1β (**A**), IL-6 (**B**), and TNF-α (**C**) and was detected by quantitative real time PCR. Values are shown as means ± SD (*n* = 3). *n*, the number of replicates. Different letters (a, b, ab, and c) indicate statistical significance.

rLysG1 exhibited relatively strong binding to Gram-negative bacteria, including those, such as the Vibrios, that were immune to the killing of rLysG1, whereas the binding activity of rLysG1 to Gram-positive bacteria was comparatively much lower or barely detectable (Figure 3A). Microscopy confirmed localization of rLysG1 on the cell surface following incubation of rLysG1 with the bacteria (Figure 3B). In contrast, no binding of rTrx, a control protein, to bacteria was detected (Figure 3B). Consistent with these observations, rLysG1 bound efficiently to the cell wall components of Gram-negative bacteria (LPS and lipid A), and much weakly to that of Gram-positive bacteria (PGN) (Figure 3C). The presence of Cu^2+^, however, impaired the binding of rLysG1 to bacteria, LPS and lipid A (Figure S3). Interaction with exogenous LPS significantly inhibited the ability of rLysG1 to bind and kill bacteria in a manner that depended on the concentration of LPS (Figure 3D). To examine whether the LPS-binding capacity of rLysG1 would have any effect on LPS-induced inflammatory response, RAW264.7 cells were treated with LPS and examined for the expression of IL-1β, IL-6, and TNF-α. All these cytokines were significantly suppressed by rLysG1 in a dose-dependent manner (Figure 4). 

### 2.4. rLysG1 Kills Bacteria by Disrupting Bacterial Membrane

**Figure 5:**
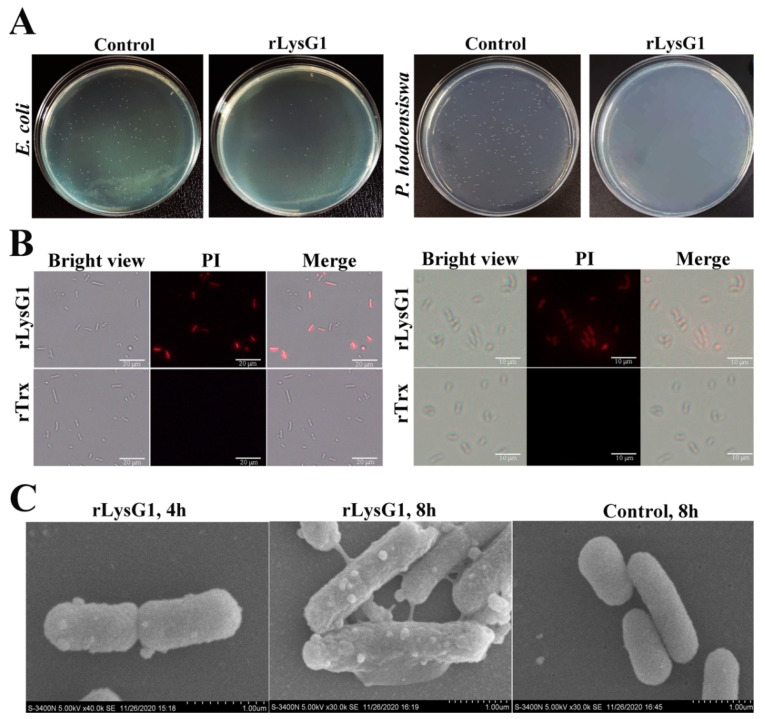
The effect of rLysG1 on bacterial cell membrane integrity. (**A**) *Escherichia coli* and *Psedoalteromonas hodoensiswas* were incubated with rLysG1 or PBS (control) for 4 h, and bacterial survival was observed on plates. (**B**) *E. coli* (**left**) and *P. hodoensiswas* (**right**) were incubated with rLysG1 or rTrx for 4 h. The cells were stained with PI and observed with a fluorescence microscope. (**C**) *E. coli* was incubated with or without (control) rLysG1 for 4 h or 8 h and then observed with a scanning electron microscope.

When grown in the presence of rLysG1, *E. coli* and *P. hodoensiswas* formed significantly reduced numbers of colonies compared to growth in the absence of rLysG1 (Figure 5A), suggesting that most of the parental bacterial cells were killed by rLysG1. Following treatment with rLysG1, the bacterial cells were stained positive by propidium iodide (PI) (Figure 5B), which passes into the cells only when the cellular membrane is permeabilized or damaged. Scanning electron microscope (SEM) revealed time-dependent change in the morphology of rLysG1-treated bacterial cells. The cellular membrane became rough and formed blebs that increased in number and size with time (Figure 5C). The cells were eventually completely disrupted in structure. 

### 2.5. The Bacteriolytic Activity of rLysG1 Depends on Specific Structural Motifs 

**Figure 6:**
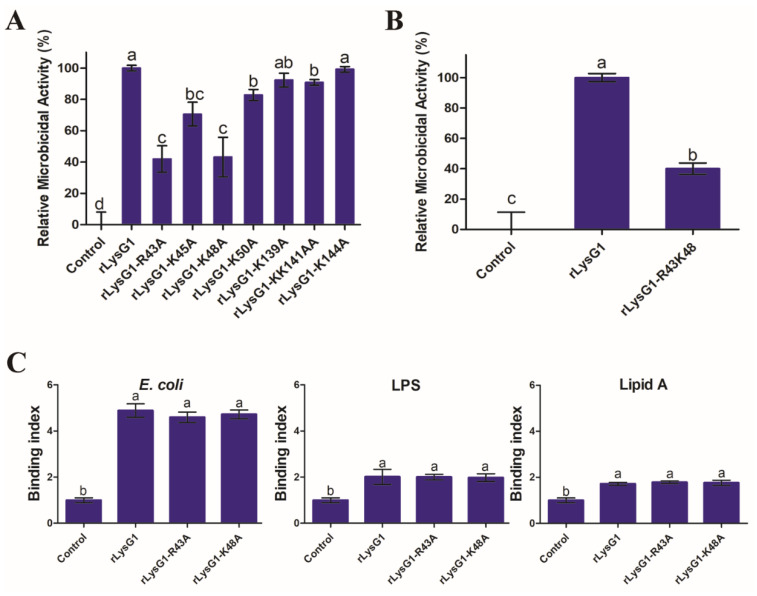
The bacterial binding and killing activities of rLysG1 variants. (**A**) *Escherichia coli* was incubated with or without (control) rLysG1 variants for 4 h, and the bactericidal activity of rLysG1 was then determined. (**B**) *E. coli* was incubated as above with or without (control) rLysG1 or rLysG1-R43K48, and the bactericidal activity of rLysG1 was then determined. (**C**) rLysG1 variants and rTrx were incubated with *E. coli*, LPS, or lipid A for 1 h, and the relative binding of the protein to bacteria/cell wall component was then determined. Values are shown as means ± SD (*n* = 3). *n*, the number of replicates. For all panels, different letters (a, b, ab, c, bc and d) indicate statistical significance.

To find the structural feature that might be important to the activity of LysG1, recombinant proteins of LysG1 mutants were prepared, i.e., rLysG1-R43A, rLysG1-K45A, rLysG1-K48A, rLysG1-K50A, rLysG1-K139A, rLysG1-K141A-K142A, and rLysG1-K144A, which bear alanine substitution at the basic residues located in the α3, 4, and 7 helixes, i.e., R43, K45, K48, K50, K139, K141, K142, and K144, respectively. Of these mutants, rLysG1-R43A and rLysG1-K48A were markedly and significantly reduced in bactericidal activity compared to rLysG1, whereas the other mutants were little or not affected (Figure 6A). The bactericidal activity of the mutant rLysG1-R43A-K48A, which bears double alanine substations at R43 and K48, was only 40% of that of rLysG1 (Figure 6B). Binding analysis showed that both rLysG1-R43A and rLysG1-K48A bound to *E. coli*, LPS, and LTA as efficiently as rLysG1 (Figure 6C).

## 3. Discussion 

In the present study, we identified a g-type lysozyme, LysG1, from the *Rimicaris* shrimp found in a hydrothermal vent of Manus Basin. LysG1 exhibits the conserved carbohydrate binding site and catalytic site of g-type lysozyme, but apparently lacks a signal peptide. Previous reports showed that almost all g-type lysozymes in birds and mammals contain predicted signal sequences for secretion, whereas most fish g-type lysozymes and some invertebrate g-type lysozymes do not have the characteristic secretion signals, suggesting that they may function within the cells [14,18,36]. In invertebrate, studies on g-type lysozyme are very limited, chiefly in *Haliotis discus discus*, *Chlamys farreri*, and *Ciona* [16,18,20]. In fact, g-type lysozyme was even long considered to be absent in arthropods based on the absence of g-type lysozyme-encoding genes in the available genomes of the most abundant class of arthropods—the insect [37]. Indeed, a search of NCBI database yielded only two deposited g-type lysozyme sequences from crustacean, i.e., *C. rogercresseyi* and *M. nipponense*. LysG1 was phylogenetically clustered together with the g-type lysozyme of *M. nipponense*, and differs from the two crustacean lysozymes by 47% and 46.2%, respectively, in sequence. These results indicate that LysG1 has evolved differently and acquired new sequence features that are distinct from that of other crustaceans.

The antimicrobial mechanism of lysozymes can be classified as the enzyme-dependent mode and the non-enzymatic mode [14]. Most vertebrate and invertebrate lysozymes are active muramidases and can act upon a broad spectrum of bacteria [20,38,39,40,41,42]. A small group of lysozymes exert antibacterial effects in a muramidase-independent manner. For example, a T4 lysozyme and two i-type lysozymes from red swamp crayfish were able to kill bacteria independent of enzyme activity [5,43]. In our study, rLysG1 could not degrade PGN and, consistently, showed no evident effect on the survival and growth of Gram-positive bacteria. In contrast, rLysG1 effectively killed some Gram-negative bacteria. This property implies that rLysG1 may be used as a bactericidal agent against infections caused only by Gram-negative bacterial pathogens, such as *E. coli*. In agreement with these observations, rLysG1 bound more efficiently to Gram-negative bacteria, as well as their cell wall components, than to Gram-positive bacteria, and pre-binding of free LPS diminished the bacterial killing effect of rLysG1. These results suggest that rLysG1 bound target bacteria via interaction with LPS, which is a prerequisite for the bactericidal activity of rLysG1. 

A number of studies have demonstrated that pH, temperature, and metal ions can influence the activity of some lysozymes [38,39,40,44,45]. The structure of lysozyme can change greatly at different pH [46]. Metal ions, such as Ca^2+^ and Mn^2+^, are known to affect the activity of lysozymes [44,45]. Similarly, in our study, we found that the bactericidal activity of rLysG1 was optimal only at certain pH and temperature under the specified experimental conditions. Cu^2+^ and Zn^2+^ had a significant inhibitory effect on rLysG1, which may not be due to the charged status of these ions, since other divalent ions failed to produce any effect. Together, these results indicate that the antibacterial activity of rLysG1 is regulated by multiple factors. These factors, such as metal ions, pH, and temperature, may be used to manipulate the working effect of rLysG1. 

A study of hen egg white lysozyme (HEWL) showed that the enzyme rapidly increased the permeability of the outer membrane of *E. coli* due to large size pore formation [3]. It also observed a direct delayed activity of lysozyme against the inner membrane, but without evidence of perforations [3]. In our study, we found that bacteria treated with rLysG1 were stained positive by PI, suggesting cellular membrane permealization. In support of this observation, scanning electron microscopy revealed time-dependent disruption of the surface structure of rLysG1-treated bacteria. Similar membrane damages by lysozymes or lysozyme-derived antimicrobial peptides have been reported previously [47,48,49]. The membrane lytic effect of some non-enzymatic lysozymes was thought to be related to the extreme cationic charges of the enzymes [6]. In the case of LysG1, it is rich in arginine and lysine residues and exhibits a high pI of 9.06. Mutation of two of the basic amino acid residues, i.e., R43 and K48, located in the α3 and 4 helixes caused significant reductions in the activity of LysG1, implying an essential role of these residues in the interaction with target bacteria.

In addition to its direct bacteriolytic effect, lysozyme also plays a part in limiting inflammation [9]. During the infection of bacterial pathogens, lysozyme can reduce the pathogen-induced inflammation by inhibiting the growth of the bacteria [50]. For the lysozymes with muramidase activity, they can digest bacterial peptidoglycan to reduce the production of anaphylatoxins, hence limiting the inflammatory response [51]. In our study, rLysG1 was found to bind not only LPS but also lipid A, the endotoxin that is essentially responsible for the toxic effect of Gram-negative bacteria. In accordance with this property, rLysG1 effectively reduced the expression of inflammatory genes in LPS-stimulated macrophages. Inflammation is an important immune response against pathogen infection. However, excessive inflammation can cause tissue damage and disease development. Hence, the attenuating effect of LysG1 on LPS-induced inflammatory response may be beneficial to the host. 

## 4. Materials and Methods

### 4.1. Bacteria Strains and Culture Conditions

The Gram-positive bacteria *Staphylococcus aureus*, *Streptococcus iniae*, *Micrococcus luteus* and *Bacillus subtilis* and the Gram-negative bacteria *Pseudomonas fluorescens*, *Escherichia coli*, *Edwardsiella tarda*, *Vibrio harveyi*, and *Vibrio anguillarum* have been reported previously [34]. The Gram-positive bacteria *Bacillus thuringiensis*, *Bacillus mobilis*, and *Bacillus toyonensis*, and the Gram-negative bacteria *Psedoalteromonas hodoensis*, *Vibrio neocaledonicus*, *Alteromonas abrolhosensis*, and *Pseudidiomarina donghaiensisare* were isolated from deep sea environments. *S. iniae* was cultured in TSB medium (Hopobio, Qingdao, China) at 28 °C. The deep-sea bacteria were cultured in marine 2216E medium at 28 °C. All other bacteria were culture in Luria-Bertani broth (LB) medium at 28 °C (for *B. subtilis, P. fluorescens, E. tarda, V. harveyi*, and *V. anguillarum*) or 37 °C (for *M. luteus, S. aureus* and *E. coli*).

### 4.2. Sequence and Phylogenetic Analysis

The sequence of LysG1 was obtained from the transcriptome of Alvinocarididae shrimp [28]. The sequence was analyzed with DNAMAN 6.0 (Lynnon Biosoft, San Ramon, CA, USA). Sequence BLAST was performed using the Protein BLAST algorithm of the NCBI (http://www.ncbi.nlm.nih.gov/blast (accessed on 3 December 2020)). Signal peptide search was performed using the SignalP program (http://www.cbs.dtu.dk/services/SignalP (accessed on 3 December 2020)). Multiple sequence alignment was performed with the ClustalX software version 2.1. The output picture was generated using ESPript 3.0 (https://espript.ibcp.fr/ESPript/ESPript/index.php (accessed on 1 December 2020)). The structure modeling of LysG1 was predicted with the SWISS-MODEL (https://swissmodel.expasy.org/interactive (accessed on 14 December 2020)). The phylogenetic tree was constructed using neighbour-joining method with the MEGA software version 7.0.26, and the reliability of the tree was assessed by bootstrapping (1000 replications).

### 4.3. Plasmid Construction and Protein Purification

To construct the plasmid pET28a-LysG1, which expresses recombinant LysG1 (rLysG1) with a His-tag, the coding sequence of LysG1 was inserted into the expression plasmid pET28a at the NdeI/XhoI sites, resulting in pET28a-LysG1. The pET28a-LysG1 and pET32a (for purification of His-tagged Trx as a control protein) were separately introduced into *Escherichia coli* BL21 (DE3) by transformation. The transformants were cultured in LB medium at 37 °C to OD_600_ of 0.4, and then 0.04 mM Isopropyl-β-D-Thiogalactopyranoside (IPTG) was added. The culture was continued at 16 °C for 15 h with shaking (100 rpm). The cells were then harvested by centrifugation. The proteins with His-tag were purified with nickel-nitrilotriacetic acid (Ni-NTA) columns (QIAGEN, Germantown, USA) as described previously [52]. The recombinant LysG1 and Trx eluates were dialyzed extensively against PBS (pH 7.2) supplemented with 10% glycerol at 4 °C for 24 h. The purified proteins were analyzed using sodium dodecyl sulfate-polyacrylamide gel electrophoresis (SDS-PAGE). To prepare the mutant LysG1 variants, the plasmids expressing the mutants were constructed by inserting the coding sequences of LysG1 bearing alanine substitution at R43, K45, K48, K50, K139, KK141, K144 and R43K48 into pET28a as above. The mutant proteins were purified as above.

### 4.4. Muramidase Activity 

The DNS (3, 5-Dinitrosalicylic acid) method was used to detect the muramidase activity of rLysG1 as reported previously [53]. Briefly, a standard curve of N-acetylglucosamine reaction with DNS was generated as reported previously [53]. To determine the muramidase activity of rLysG1, 16 μM of rLysG1 or lysozyme (Sigma, St Louis, MO, USA) (positive control) was added to PBS buffer containing 10 mg/mL peptidoglycan (PGN). After incubation at 37 °C for 4 h, DNS (Sangon Biotech, Shanghai, China) was added to terminate the reaction, and the Eppendorf tube containing the sample was placed in boiling water for 5 min for color to develop. The solution was centrifuged at 6000 rpm for 10 min, and the optical density of the supernatant was measured at 520 nm.

### 4.5. Bactericidal Activity 

To determine the minimal bactericidal concentration (MBC) of rLysG1, bacteria were grown to logarithmic phase and resuspended in PBS to 10^3^ CFU/mL with PBS. Then 0.5 μM to 16 μM (2-fold dilution) of rLysG1 was added to 200 μL bacterial suspension. PBS was added to the control bacterial suspension. The samples were incubated at 37 °C for 4 h, and bacterial survival was determined by plate count [34]. To examine the effect of temperature and pH on the bactericidal activity of rLysG1, *E. coli* was incubated with or without (control) rLysG1 (8 μM) at different temperatures (4, 16, 37 and 42 °C) or pH (5.6, 6.0, 6.4, 7.0, 7.6 and 8.0) for 4 h. To examine the effect of metal ions, *E. coli* was incubated with or without rLysG1 (8 μM) in PBS containing 100 μM FeCl_2_, CuCl_2_, CaCl_2_, MgCl_2_, MnCl_2_, CoCl_2_, or ZnCl_2_. To determine the effect of LPS on the bactericidal activity of rLysG1, rLysG1 (8 μM) was pre-incubated with LPS (Sigma, St Louis, MO, USA) (0.4, 0.8, or 2 mg/mL) at 37 °C for 1 h. *E. coli* was then added to the mixture, and the mixture was incubated at 37 °C for 4 h. To examine the bactericidal activity of the rLysG1 mutants, *E. coli* was incubated with or without each of the rLysG1 mutants (8 μM) at 37 °C for 4 h. In all cases, bacterial survival was determined as above. 

### 4.6. Binding of rLysG1 to Bacteria and Bacterial Cell Wall Components

Bacterial cells were placed into a 96-well microtiter plate containing coating buffer (1.59 g Na_2_CO_3_ and 2.93 g NaHCO_3_/L, pH 9.6) at the final concentration of 10^8^ CFU/mL. The plate was incubated at 4 °C overnight. LPS or PGN (Sigma, St Louis, MO, USA) dissolved in the coating buffer to 200 μg/mL was added to a 96-well microtiter plate, followed by incubation at 4 °C overnight. Lipid A (Sigma, St Louis, MO, USA) (200 μg/mL in methanol) was added to a 96-well microtiter plate, and the plate was left to dry at room temperature for 2 h. All the plates were washed 3 times with PBST (PBS with 0.05% Tween 20) and blocked with 150 µL 5% skim milk powder (Solarbio, Beijing, China) at 37 °C for 1 h. The plates were washed as above, and different concentrations of rLysG1 or PBS was added to the wells. The plates were incubated at 37 °C for 1 h and washed as above. The HRP-conjugated mouse anti-His antibody (Abcam, Cambridge, MA, USA) (1/1000 dilution) was added to the plates. The plates were incubated at 37 °C for 1 h and washed as above. Color development was performed using TMB substrate solution (Tiangen, Beijing, China) and terminated by adding ELISA stop solution (Solarbio, Beijing, China). Finally the absorbance at 450 nm was measured. To examine binding of rLysG1 to bacteria by microscopy, *E. coli* and *P. hodoensiswas* were incubated with rLysG1 or rTrx for 1 h at 37 °C and washed 5 times with PBS. Then the cells were successively treated with the mouse anti-His antibody (Abcam, Cambridge, MA, USA) (1/1000 dilution) and FITC-labeled goat anti-mouse secondary antibody (Abcam, Cambridge, MA, USA) (1/1000 dilution) for 1 h at 37 °C. After washing 5 times with PBS, the cells were observed with a fluorescence microscope (TiS/L100, Nikon, Tokyo, Japan). 

### 4.7. Examination of Bacterial Cell Membrane Integrity

*E. coli* and *P. hodoensiswas* were cultured to OD_600_ of 1.0 and resuspended in PBS to 5× 10^8^ CFU/mL. The cells were incubated with rLysG1 or rTrx for 2 h at 37 °C and washed 5 times with PBS. The cells were stained with propidium iodide (50 μg/mL) for 5 min and washed as above. The cells were then observed with a fluorescence microscope as above. For electron microscopic examination of bacterial structural change caused by rLysG1, *E. coli* and *P. hodoensiswas* were incubated with rLysG1 or rTrx and washed with PBS as above. The cells were fixed and processed as reported previously [34]. The samples were then examined with a scanning electron microscope (S-3400N, Hitachi, Tokyo, Japan).

### 4.8. Cell Culture

RAW264.7 cells were cultured in DMEM (Gibco, Big Cabin, OK, USA) supplemented with 10% fetal bovine serum (Gibco, USA) and 1% penicillin and streptomycin (Beyotime Biotechnology, Shanghai, China) at 37 °C and 5% CO_2_.

### 4.9. Quantitative Real-Time PCR (qRT-PCR)

To examine the effect of rLysG1 on LPS-induced inflammatory gene expression, LPS (1 μg/mL) (Sigma, St Louis, MO, USA) was incubated with or without rLysG1 (2, 4 or 8 μM) for 1 h at 37 °C. RAW264.7 cells were incubated with rLysG1-treated LPS or untreated LPS for 24 h at 37 °C. The expression of TNF-α, IL-6 and IL-1β was determined by qRT-PCR as reported previously [54].

## 5. Conclusions

In Summary, we identified and investigated the activity of a g-type lysozyme from deep-sea shrimp. rLysG1 killed selectively Gram-negative bacteria in a non-enzymatic mode by damaging bacterial membrane. The bactericidal activity of rLysG1 was regulated by multiple factors and required the integrity of the α3 and 4 helixes of the protein. The results of our study provide insights into the characteristics and function of g-type lysozyme in crustacean and deep-sea organisms.

## Figures and Tables

**Table 1:** The minimal bactericidal concentration (MBC) of rLysG1 against Gram-positive and Gram-negative bacteria.

Microorganisms	MBC (µM)
Gram-negative bacteria	
*Pseudomonas fluorescens*	8
*Escherichia coli*	4
*Edwardsiella tarda*	16
*Vibrio harveyi*	-
*Vibrio anguillarum*	-
Gram-positive bacteria	
*Staphylococcus aureus*	-
*Streptococcus iniae*	-
*Micrococcus luteus*	-
*Bacillus subtilis*	-
Gram-negative bacteria (Deep-sea)	
*Psedoalteromonas hodoensis*	4
*Vibrio neocaledonicus*	-
*Alteromonas abrolhosensis*	-
*Pseudidiomarina donghaiensis*	-
Gram-positive bacteria (Deep-sea)	
*Bacillus thuringiensis*	-
*Bacillus mobilis*	-
*Bacillus toyonensis*	-

-: No inhibitory or bactericidal activity was detected at the tested concentration (0.5–16 µM).

## Data Availability

Not applicable.

## Supplementary Materials

The following are available online. Figure S1: SDS-PAGE analysis of rLysG1, Figure S2: Analysis of the potential digestive effect of rLysG1 on peptidoglycan (PGN), Figure S3: The effect of Cu^2+^ on the binding of rLysG1 to bacteria and bacterial components, Table S1: Primers used in this study.

## References

[1] Phillips D.C. (1966). The Three-Dimensional Structure of an Enzyme Molecule. Sci. Am..

[2] De-La-Re-Vega E., Garcia-Galaz A., Diaz-Cinco M.E., Sotelo-Mundo R.R. (2006). White shrimp (*Litopenaeus vannamei*) recombinant ly-sozyme has antibacterial activity against Gram negative bacteria: *Vibrio alginolyticus*, *Vibrio parahemolyticus* and *Vibrio cholerae*. Fish Shellfish Immunol..

[3] Derde M., Lechevalier V., Guérin-Dubiard C., Cochet M.-F., Jan S., Baron F., Gautier M., Vié V., Nau F. (2013). Hen Egg White Lysozyme Permeabilizes Escherichia coli Outer and Inner Membranes. J. Agric. Food Chem..

[4] Ibrahim H.R., Matsuzaki T., Aoki T. (2001). Genetic evidence that antibacterial activity of lysozyme is independent of its catalytic function. FEBS Lett..

[5] Zhang H.-W., Sun C., Sun S.-S., Zhao X.-F., Wang J.-X. (2010). Functional analysis of two invertebrate-type lysozymes from red swamp crayfish, *Procambarus clarkii*. Fish Shellfish Immunol..

[6] Laible N.J., Germaine G.R. (1985). Bactericidal activity of human lysozyme, muramidase-inactive lysozyme, and cationic polypeptides against *Streptococcus sanguis* and *Streptococcus faecalis*: Inhibition by chitin oligosaccharides. Infect. Immun..

[7] Masschalck B., Michiels C.W. (2003). Antimicrobial properties of lysozyme in relation to foodborne vegetative bacteria. Crit. Rev. Microbiol..

[8] Gordon L., Douglas S.D., Kay N.E., Yamada O., Osserman E.F., Jacob H.S. (1979). Modulation of neutrophil function by lysozyme. Potential negative feedback system of inflammation. J. Clin. Investig..

[9] Ragland S.A., Criss A.K. (2017). From bacterial killing to immune modulation: Recent insights into the functions of lysozyme. PLoS Pathog..

[10] Lee M., Kovacs-Nolan J., Yang C., Archbold T., Fan M.Z., Mine Y. (2009). Hen Egg Lysozyme Attenuates Inflammation and Modulates Local Gene Expression in a Porcine Model of Dextran Sodium Sulfate (DSS)-Induced Colitis. J. Agric. Food Chem..

[11] Ogundele M.O. (1998). A novel anti-inflammatory activity of lysozyme: Modulation of serum complement activation. Mediat. Inflamm..

[12] Saurabh S., Sahoo P.K. (2008). Lysozyme: An important defence molecule of fish innate immune system. Aquac. Res..

[13] Canfield R.E., McMurry S. (1967). Purification and characterization of a lysozyme from goose egg white. Biochem. Biophys. Res. Commun..

[14] Callewaert L., Michiels C.W. (2010). Lysozymes in the animal kingdom. J. Biosci..

[15] Irwin D.M., Gong Z.M. (2003). Molecular Evolution of Vertebrate Goose-Type Lysozyme Genes. J. Mol. Evol..

[16] Bathige S., Umasuthan N., Whang I., Lim B.-S., Jung H.-B., Lee J. (2013). Evidences for the involvement of an invertebrate goose-type lysozyme in disk abalone immunity: Cloning, expression analysis and antimicrobial activity. Fish Shellfish Immunol..

[17] He C., Yu H., Liu W., Su H., Shan Z., Bao X., Li Y., Fu L., Gao X. (2012). A goose-type lysozyme gene in Japanese scallop (*Mizuhopecten yessoensis*): cDNA cloning, mRNA expression and promoter sequence analysis. Comp. Biochem. Physiol. Part B Biochem. Mol. Biol..

[18] Nilsen I.W., Myrnes B., Edvardsen R.B., Chourrout D. (2003). Urochordates carry multiple genes for goose-type lysozyme and no genes for chicken- or invertebrate-type lysozymes. Cell. Mol. Life Sci..

[19] Wang Q., Zhang L., Zhao J., You L., Wu H. (2012). Two goose-type lysozymes in *Mytilus galloprovincialis*: Possible function diversifi-cation and adaptive evolution. PLoS ONE.

[20] Zhao J., Song L., Li C., Zou H., Ni D., Wang W., Xu W. (2007). Molecular cloning of an invertebrate goose-type lysozyme gene from Chlamys farreri, and lytic activity of the recombinant protein. Mol. Immunol..

[21] Cai S., Zhang Y., Wu F., Wu R., Yang S., Li Y., Xu Y. (2019). Identification and functional characterization of a c-type lysozyme from *Fenneropenaeus penicillatus*. Fish Shellfish Immunol..

[22] Hikima S., Hikima J., Rojtinnakorn J., Hirono I., Aoki T. (2003). Characterization and function of kuruma shrimp lysozyme pos-sessing lytic activity against Vibrio species. Gene.

[23] Liu H.-T., Wang J., Mao Y., Liu M., Niu S.-F., Qiao Y., Su Y.-Q., Wang C.-Z., Zheng Z.-P. (2016). Identification and expression analysis of a new invertebrate lysozyme in Kuruma shrimp (*Marsupenaeus japonicus*). Fish Shellfish Immunol..

[24] Liu Y., Zhang Y.H., Li T., Cao X.T., Zhou Y., Yuan J.F., Gu Z.M., Lan J.F. (2018). PcLys-i3, an invertebrate lysozyme, is involved in the an-tibacterial immunity of the red swamp crayfish, *Procambarus clarkii*. Dev. Comp. Immunol..

[25] Sotelo-Mundo R.R., Islas-Osuna M.A., De-La-Re-Vega E., Hernández-López J., Vargas-Albores F., Yepiz-Plascencia G. (2003). cDNA cloning of the lysozyme of the white shrimp *Penaeus vannamei*. Fish Shellfish Immunol..

[26] Supungul P., Rimphanitchayakit V., Aoki T., Hirono I., Tassanakajon A. (2010). Molecular characterization and expression analysis of a c-type and two novel muramidase-deficient i-type lysozymes from *Penaeus monodon*. Fish Shellfish Immunol..

[27] Ye X., Gao F.Y., Zheng Q.M., Bai J.J., Wang H., Lao H.H., Jian Q. (2009). Cloning and characterization of the tiger shrimp lysozyme. Mol. Biol. Rep..

[28] Zhang J., Sun Q.-L., Luan Z.-D., Lian C., Sun L. (2017). Comparative transcriptome analysis of Rimicaris sp. reveals novel molecular features associated with survival in deep-sea hydrothermal vent. Sci. Rep..

[29] Little C., Vrijenhoek R.C. (2003). Are hydrothermal vent animals living fossils?. Trends Ecol. Evol..

[30] Van Dover C.L., German C.R., Speer K.G., Parson L.M., Vrijenhoek R.C. (2002). Evolution and biogeography of deep-sea vent and seep invertebrates. Science.

[31] Martin J.W., Haney T.A. (2005). Decapod crustaceans from hydrothermal vents and cold seeps: A review through 2005. Zool. J. Linn. Soc..

[32] Hernández-Ávila I., Cambon-Bonavita M.-A., Pradillon F. (2015). Morphology of First Zoeal Stage of Four Genera of Alvinocaridid Shrimps from Hydrothermal Vents and Cold Seeps: Implications for Ecology, Larval Biology and Phylogeny. PLoS ONE.

[33] Liu X.L., Ye S., Cheng C.Y., Li H.W., Lu B., Yang W.J., Yang J.S. (2019). Identification and characterization of a symbiotic agglutina-tion-related C-type lectin from the hydrothermal vent shrimp Rimicaris exoculata. Fish Shellfish Immunol..

[34] Wang Y., Zhang J., Sun Y., Sun L. (2021). A Crustin from Hydrothermal Vent Shrimp: Antimicrobial Activity and Mechanism. Mar. Drugs.

[35] Le Bloa S., Boidin-Wichlacz C., Cueff-Gauchard V., Rosa R.D., Cuvillier-Hot V., Durand L., Methou P., Pradillon F., Cam-bon-Bonavita M.A., Tasiemski A. (2020). Antimicrobial peptides and ectosymbiotic relationships: Involvement of a novel type iia crustin in the life Cycle of a deep-sea vent shrimp. Front. Immunol..

[36] Larsen A.N., Solstad T., Svineng G., Seppola M., Jørgensen T.Ø. (2009). Molecular characterisation of a goose-type lysozyme gene in Atlantic cod (*Gadus morhua* L.). Fish Shellfish Immunol..

[37] Van Herreweghe J.M., Michiels C.W. (2012). Invertebrate lysozymes: Diversity and distribution, molecular mechanism and in vivo function. J. Biosci..

[38] Buonocore F., Randelli E., Trisolino P., Facchiano A., de Pascale D., Scapigliati G. (2014). Molecular characterization, gene structure and antibacterial activity of a g-type lysozyme from the European sea bass (*Dicentrarchus labrax* L.). Mol. Immunol..

[39] Nilojan J., Bathige S., Kugapreethan R., Thulasitha W., Nam B.-H., Lee J. (2017). Molecular, transcriptional and functional insights into duplicated goose-type lysozymes from *Sebastes schlegelii* and their potential immunological role. Fish Shellfish Immunol..

[40] Yang H., Liu R., Cui D., Liu H., Xiong D., Liu X., Wang L. (2017). Analysis on the expression and function of a chicken-type and goose-type lysozymes in Chinese giant salamanders *Andrias davidianus*. Dev. Comp. Immunol..

[41] Yin Z., He J., Deng W., Chan S. (2003). Molecular cloning, expression of orange-spotted grouper goose-type lysozyme cDNA, and lytic activity of its recombinant protein. Dis. Aquat. Org..

[42] Zhang S.H., Zhu D.D., Chang M.X., Zhao Q.P., Jiao R., Huang B., Fu J.P., Liu Z.X., Nie P. (2012). Three goose-type lysozymes in the gastropod *Oncomelania hupensis*: cDNA sequences and lytic activity of recombinant proteins. Dev. Comp. Immunol..

[43] Düring K., Porsch P., Mahn A., Brinkmann O., Gieffers W. (1999). The non-enzymatic microbicidal activity of lysozymes. FEBS Lett..

[44] Gorini L., Felix F. (1953). Influence of manganese on the stability of lysozyme. I. Influence of manganese on the rate of irreversible inactivation of lysozyme by heat. Biochim. Biophys. Acta..

[45] Kuroki R., Taniyama Y., Seko C., Nakamura H., Kikuchi M., Ikehara M. (1989). Design and creation of a Ca2+ binding site in human lysozyme to enhance structural stability. Proc. Natl. Acad. Sci. USA.

[46] Bonincontro A., De Francesco A., Onori G. (1998). Influence of pH on lysozyme conformation revealed by dielectric spectroscopy. Colloids Surf. B Biointerfaces.

[47] Pellegrini A., Thomas U., Wild P., Schraner E., Von Fellenberg R. (2000). Effect of lysozyme or modified lysozyme fragments on DNA and RNA synthesis and membrane permeability of *Escherichia coli*. Microbiol. Res..

[48] Thammasirirak S., Pukcothanung Y., Preecharram S., Daduang S., Patramanon R., Fukamizo T., Araki T. (2010). Antimicrobial peptides derived from goose egg white lysozyme. Comp. Biochem. Physiol. Part C Toxicol. Pharmacol..

[49] Wild P., Gabrieli A., Schraner E.M., Pellegrini A., Thomas U., Frederik P.M., Stuart M.C., Von Fellenberg R. (1997). Reevaluation of the effect of lysoyzme on *Escherichia coli* employing ultrarapid freezing followed by cryoelectronmicroscopy or freeze substitu-tion. Microsc. Res. Tech..

[50] Ganz T., Gabayan V., Liao H.-I., Liu L., Oren A., Graf T., Cole A.M. (2003). Increased inflammation in lysozyme M–deficient mice in response to *Micrococcus luteus* and its peptidoglycan. Blood.

[51] Riber U., Espersen F., Wilkinson B.J., Kharazmi A. (1990). Neutrophil chemotactic activity of peptidoglycan. A comparison between *Staphylococcus-Aureus* and *Staphylococcus-Epidermidis*. APMIS.

[52] Jiang S., Gu H., Zhao Y., Sun L. (2019). Teleost Gasdermin E Is Cleaved by Caspase 1, 3, and 7 and Induces Pyroptosis. J. Immunol..

[53] Guan B., Ding Y., Xie L., Yan L. (1999). The Modification of the DNS Method for the Determination of Reducing Sugar. J. WuXi Univ. Light Ind..

[54] Zhang T., Jiang S., Sun L. (2020). A Fish Galectin-8 Possesses Direct Bactericidal Activity. Int. J. Mol. Sci..

