# Supplementary material for: A g-Type Lysozyme from Deep-Sea Hydrothermal Vent Shrimp Kills Selectively Gram-Negative Bacteria
Source: Molecules. 2021 Dec 16;26(24):7624. doi: 10.3390/molecules26247624 (PMC8707215; doi:10.3390/molecules26247624)
Supplement: Supplementary file 1 [file molecules-26-07624-s001.zip › molecules-1481940-supplementary.pdf]

Supplementary data

Table S1. Primers used in this study.

Primer	Sequence (5’-3’)
R43-F	GACGGATCTTCAAGCAATGAAAGGCTAC
R43-R	GTAGCCTTTCATTGCTTGAAGATCCGTC
K45-F	CGGATCTTCAAAGGATGGCAGGCTACAAGCAAAAG
K45-R	CTTTTGCTTGTAGCCTGCCATCCTTTGAAGATCCG
K48-F	CAAAGGATGAAAGGCTACGCACAAAAGATCTCCAGTGTTG
K48-R	CAACACTGGAGATCTTTTGTGCGTAGCCTTTCATCCTTTG
K50-F	GAAAGGCTACAAGCAAGCAATCTCCAGTGTTGGGAC
K50-R	GTCCCAACACTGGAGATTGCTTGCTTGTAGCCTTTC
K139-F	CCTTCCAAGGCCTGTCCGCAGAGAAGAAGTTGAAAG
K139-R	CTTTCAACTTCTTCTCTGCGGACAGGCCTTGGAAGG
KK141-F	CCAAGGCCTGTCCAAGGAGGCAGCATTGAAAGCTGGGATAGCAG
KK141-R	CTGCTATCCCAGCTTTCAATGCTGCCTCCTTGGACAGGCCTTGG
K144-F	CAAGGAGAAGAAGTTGGCAGCTGGGATAGCAGC
K144-R	GCTGCTATCCCAGCTGCCAACTTCTTCTCCTTG
TNF-α-F	ACTGAACTTCGGGGTGATCG
TNF-α-R	AGGGTCTGGGCCATAGAACT
IL6-F	AGCCAGAGTCCTTCAGAGAGAT
IL6-R	GAGAGCATTGGAAATTGGGGT
IL-1β-F	TGCCACCTTTTGACAGTGATG
IL-1β-R	TGTGCTGCTGCGAGATTTGA
β-actin-F	CTGCCGCATCCTCTTCCTC
β-actin-R	GGAAAAGAGCCTCAGGGCAT

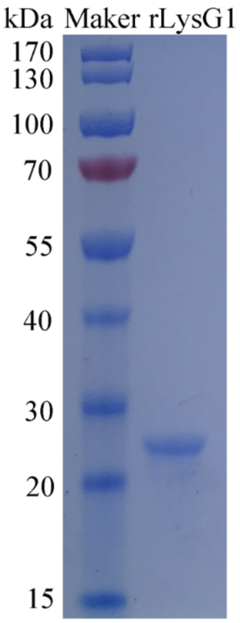

Figure S1. SDS-PAGE analysis of rLysG1. Purified rLysG1 was analyzed by SDS-PAGE and viewed after staining with Coomassie brilliant blue R-250.

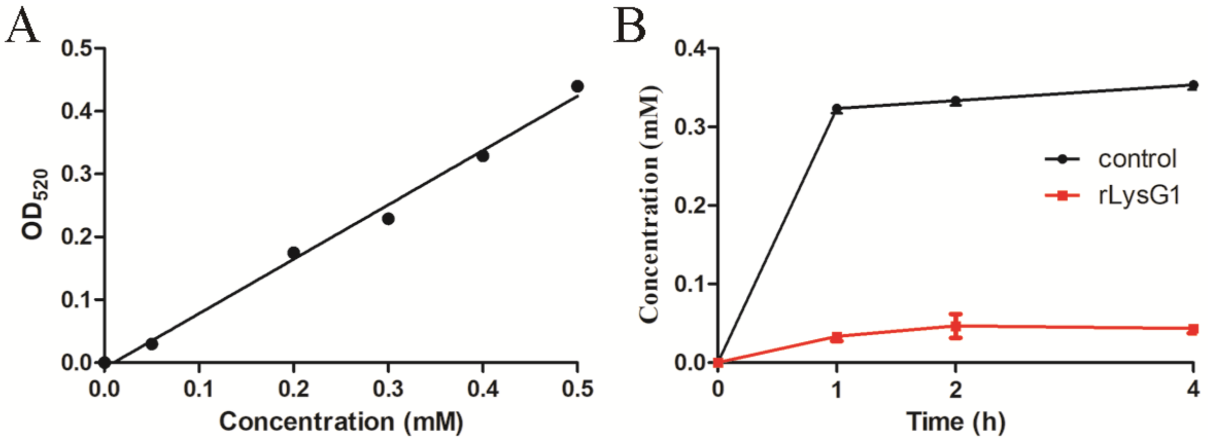

Figure S2. Analysis of the potential digestive effect of rLysG1 on peptidoglycan (PGN). (**A**) The standard curve of N-acetylglucosamine. Different concentrations of N-acetylglucosamine were incubated with 3, 5-Dinitrosalicylic acid (DNS) reagent, and the absorbance was measured at OD_{520}. (**B**) The release of N-acetylglucosamine in PGN after incubation with a commercial lysozyme (control) or rLysG1 was measured by the above DNS method at different time. Values are shown as means ± SD (*n* = 3). N, the number of replicates.

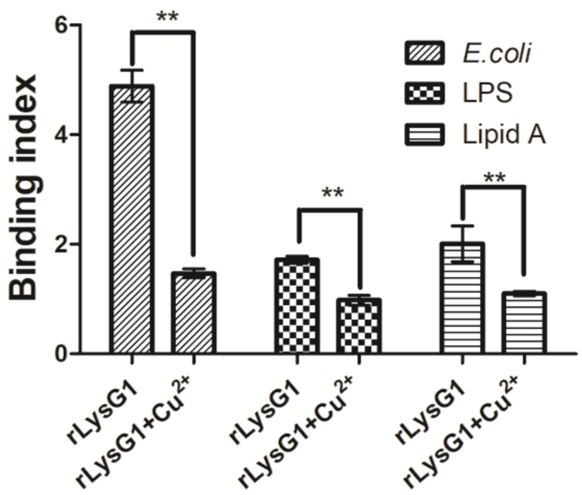

Figure S3. The effect of Cu^{2+} on the binding of rLysG1 to bacteria and bacterial components. rLysG1 was incubated with *Escherichia coli*, LPS, or lipid A in the presence or absence of Cu^{2+}, and the binding of rLysG1 was detected by ELISA. Values are shown as means ± SD (n = 3). N, the number of replicates.**, $p < 0.01$.
